# Influence of Filler Materials on Wettability and Mechanical Properties of Basalt/E-Glass Woven Fabric–Reinforced Composites for Microfluidics

**DOI:** 10.3390/mi13111875

**Published:** 2022-10-31

**Authors:** Ayyappa Atmakuri, Lalitnarayan Kolli, Arvydas Palevicius, Sigita Urbaite, Giedrius Janusas

**Affiliations:** 1 Faculty of Mechanical Engineering and Design, Kaunas University of Technology, Studentu 56, 51424 Kaunas, Lithuania; 2 Faculty of Mechanical Engineering, Sir CRR College of Engineering, Eluru 534007, Andhra Pradesh, India

**Keywords:** basalt, E-glass, epoxy resin, graphite particles, wettability, mechanical properties

## Abstract

This paper presents the development of novel hybrid composites in the presence of filler particles and manufactured using a proposed new fabrication technique. The hybrid composites were fabricated using a basalt and E-glass woven fabric–reinforced epoxy resin matrix combined with graphite powder nanoparticles. Six sets of samples were fabricated using the vacuum-assisted free lamination compression molding technique. After the fabrication, wettability, mechanical properties (tensile, flexural and impact properties) and moisture properties were evaluated. Surface morphology and chemical composition of the composite samples were examined using a scanning electron microscope (SEM) and spectroscopy. The obtained results showed that the use of filler materials in hybrid composites improves the properties of hybrid composites. Basalt/E-glass hybrid composites with 10% graphite material exhibited superior mechanical properties over the other composites, with high-quality, improved adhesion and surface morphology. Thus, novel composites with the combination of exceptional properties may be integrated in the design of flexible electronics and microfluidics devices as a structural layer of the system. High flexibility and good surface tension of the designed composites makes them attractive for using the thermal imprint technique for microfluidics channel design.

## 1. Introduction

The importance of natural fibers in the development of fiber-reinforced hybrid polymer composites is increasing day by day in the modern era because it is directly associated with environmental concerns as well as with a circular economy [1,2]. The combination of natural fibers with synthetic fibers in reinforced hybrid polymer composites offers several attractive features such as light weight, condensed lifecycle costs, high adhesion properties, flexibility and superior mechanical properties [3]. Nature is a decent source for the creation of composite materials using hemp, flax, sisal, palm [4], okra, banana, wood, bamboo, jute, sugarcane, or cowhide. Thus, all these natural materials can be a portion of the regular fiber composites, providing exceptional properties and expanding the application areas in various fields [5].

In recent years, synthetic fiber-based composites have become the leading progressive composite materials for automotive, construction, marine, sporting goods, biomedical, microfluidics and other engineering applications due to their ease of availability, high strength and modulus and also good corrosion and fatigue resistance behavior [6]. Despite the advantages to the presence of natural fibers in composite materials, there are some disadvantages, such as fiber shrinkage, poor adhesion and moisture, which may have a high effect on the mechanical properties. At the same time, most synthetic fibers are brittle in nature, which may cause a catastrophic mode of failure in the composite material [7]. Synthetic fibers such as carbon, glass, mica and nylon also play a prominent role in the composite industry. Fiber-reinforced composite materials have a better strength and modulus than numerous conventional metallic materials. These composites exhibit low explicit gravity, high strength-to-weight ratio and higher modulus–weight proportions [8,9]. Moreover, natural fibers are lower in weight compared with metallic fibers and may be applied in many weight-basic applications such as those in the aviation, civil industry and automotive areas [10].

Many researchers found interest in developing hybrid composites. The reason can be attributed to the adverse effects of polymer composites on nature (environment), their high cost and other unfavorable mechanical properties [11]. The hybridization of polymer composites is known for the improvement of mechanical properties over individual fiber composites. It is obtained by joining two or more reinforcement materials under the same matrix (thermoset or thermoplastic) material. The possible combinations are natural fiber–natural fiber, natural fiber–synthetic fiber and synthetic fiber–synthetic fiber hybrid composites [12,13]. These hybrid composites have been established to overcome the drawbacks of individual fiber composites. Thus, properly configured hybrid composites produce high-quality composites and offer good mechanical properties such as strength, stiffness, flame retardance and corrosive resistance properties [14].

Among all the prominent methods for understanding the wettability properties of composite material, various droplet based techniques are used [15], such as gravitational [16,17], acoustic [18] and magnetic [19] driving methods. Among these droplet driving methods, contact angle measurement is widely popular due to its accuracy, lower operational cost and low complex procedure. The wettability of composite material is used to understand the material’s surface properties, such as its hydrophilic or hydrophobic nature, and is also used to understand the surface tension of driven droplets. Thus, the mechanical properties of thermoset and thermoplastic resin-based hybrid polymer composites with synthetic fibers, such as carbon, nylon, glass, rayon, spandex, acrylic fibers and natural fibers, such as hemp, flax, sisal, jute, bamboo and palm fibers, have been broadly investigated by many researchers. Various researchers stated that hybrid composites have superior properties over individual fiber composites. Static and dynamic mechanical properties of basalt fiber–reinforced polymer composites showed their sensitivity to the strain rate. The mechanical properties such as tensile strength, elastic modulus and failure strain were increasing quickly with strain rate when the strain rate was over 120 s^−1^ [20]. Thus, replacing glass fiber with basalt fiber in the composites enhances mechanical properties by 35–42%, i.e., compressive strength, impact energy and Young’s modulus [21], and replacing glass fiber with E-glass fiber composites gives superior tensile strength [21,22]. Moreover, when applying the plasma treatment to basalt fibers during the fabrication, the adhesion properties and surface roughness may be improved compared to those of commercial fibers [22]. The influence of waste basalt powder on rigid polyurethane foams had some influence, too. It was observed that the addition of basalt powder triggered an increase in the reactivity of the polyurethane system during the foaming process, but it caused a reduction of mechanical properties [23]. Additionally, the basalt powder can be used as an eco-friendly modifier for the epoxy resin, improving the thermo-mechanical properties for an increase in filler content [24]. The mechanical properties of composites reinforced with fabrics having the same areal density confirmed that basalt fiber composites possess an elastic modulus higher than the corresponding glass fiber composites, while their tensile strength approaches that of equivalent carbon fiber composites, and the results of fatigue behavior confirmed the better performance of basalt fiber composites than the corresponding glass fiber composites, with higher stiffness retention at low fatigue loads and better damping properties [25]. Thus, basalt and hybrid laminates with an added configuration exhibited higher impact energy absorption capacity than glass fiber composites applying hybridization with reinforcement material [26,27,28,29,30,31,32]. However, the lack of filler material in the composite fabrication may improve adhesion but significantly reduces its mechanical properties. To overcome these problems, an attempt was made in the present paper.

In this paper, basalt-fiber (BF) and E-glass-fiber (EF)-reinforced epoxy polymer composites including graphite filler material with various weight fractions (5%, 10% and 15%) were fabricated using the vacuum-assisted free lamination compression molding technique. After, the samples were allowed the heat treatment process and then tested for mechanical properties. The mechanical characterization was carried out by conducting tensile, flexural, impact, hardness and moisture analyses. The contact angle measurement technique was used to evaluate the wettability of composite materials and to understand the material’s surface properties, such as its hydrophilic or hydrophobic nature and the surface tension of driven droplets. Surface morphology and chemical composition were examined using scanning electron microscopy (SEM) and spectroscopy methods. Thus, the obtained results proved the relevance of properly configured hybrid composites possessing high quality and good mechanical and adhesion properties together with qualitative surface morphology. The obtained results proved the relevance to use novel composites in the design of microelectromechanical devices, flexible electronics and microfluidics.

## 2. Materials and Methods

### 2.1. Materials and Fabrication

Basalt (B) and E-glass (E) woven fabrics were used as a reinforcement, and graphite (G) powder nanoparticles were used as a filler material. Epoxy resin, along with hardener, was used as a matrix material. Both basalt and E-glass fabrics have the same areal density of 198 g/m^2^, and the fabrics were taken in a square shape (300 mm × 300 mm) to fabricate the composites. The mechanical properties and chemical characteristics of the materials were considered before the experimentation in order to understand the nature and behavior during the process (Table 1).

A total of six sets of hybrid composites were fabricated using the vacuum-assisted free lamination compression molding technique. In each set of composites, a total of six sheets were taken to fabricate 3 mm thickness composite plates. To make the hybrid composites with filler material, the graphite powder nanoparticles with varying weight fractions (5%, 10% and 15%) were mixed in epoxy resin for 20 min and then applied to the composite panels. The ply sequence and orientation for each composite are described in Table 2.

After degassing, a nominal pressure of 1.5 MPa was applied, and the composite panels were cured under the same pressure and vacuum for 8 h at room temperature. Then, the composite panels were taken for a heat treatment process for eliminating the moisture content, which also serves as a post-curing process for composites. The heat treatment process was carried out for 8 h at 90 °C. Total of six sets and in each five samples were developed using different materials and filler concentrations (in total 30 samples manufactured). Thus, the composite notations are considered as pure basalt (B), pure E-glass (E), basalt and E-glass (B/E), basalt and E-glass with 5% filler (B/E/G5), basalt and E-glass with 10% filler (B/E/G10) and basalt and E-glass with 15% filler material (B/E/G15) (Table 2).

### 2.2. Analytical Equipment

After the fabrication process, composites were allowed wettability testing and mechanical characterization by conducting various tests. In each set of composite groups, samples were tested according to ASTM standards (30 measurements in total). Thus, performed mechanical tests were: the flexural test, tensile test, impact test and moisture analysis. The morphological studies were carried using the scanning electron microscope (SEM) and the chemical compositions of the composite samples were tested using the SEM-EDX spectrometer.

**Figure 1:**
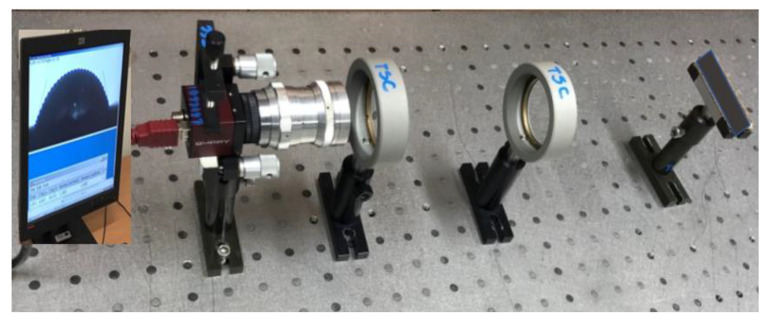
A contact angle measurement setup.

Since fabricated hybrid composites contain the filler material throughout the surface, and in future research are intended for use in flexible electronics and microfluidics devices, the wettability property of the fabricated composite surface plays a crucial role. The same test was conducted on composites without filler material for comparison purposes as well. The wettability properties of novel composite materials were measured using the contact angle measurement technique (Figure 1).

**Figure 2:**
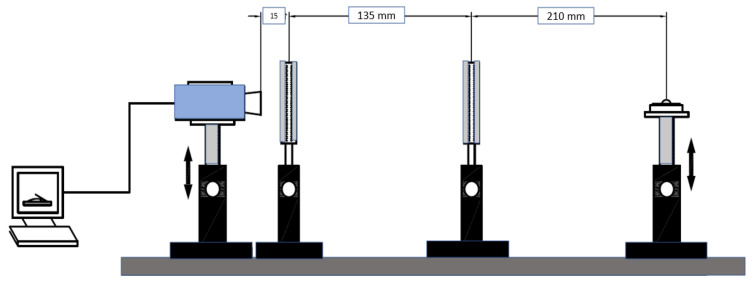
Critical distances between contact angle testing device.

The experiment was carried out in a dim laboratory space with ambient lighting. It was vital for the measurement accuracy and image analysis that the liquid drop look black, and thus the light settings were set up accordingly. The experiment was set up to eliminate any light reflections that would have tampered with the measurement (Figure 1). Additionally, precautions were taken to keep airborne contaminants such as dust and particulates from contaminating the drips. Additionally, the critical distances between the parts were kept constant during the measurements (Figure 2).

**Figure 3:**
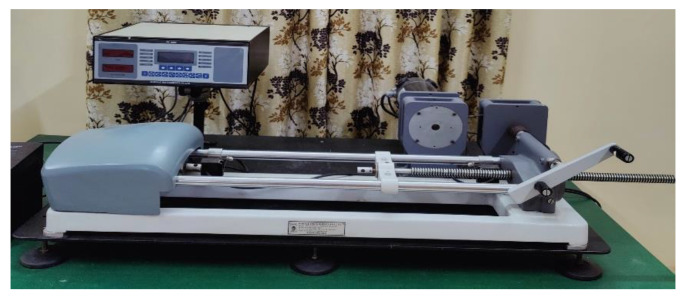
Electronic tensiometer for flexural and tensile properties testing.

Further, flexural tests were performed on fabricated samples to analyze the flexural strength and modulus, i.e., three-point bowing tests were performed on each set of composites according to ASTM D-790M-86 testing standards (total of 30 measurements). The dimensions of the samples were considered as 100 mm × 25 mm × 3 mm (length, width, thickness). The three-point bowing tests were conducted on an electronic tensiometer (METM 2000 ER-1 model (Plate II-18), provided by M/S Microtech Pune, Maharashtra, India), with outside rollers in diameter of 70 mm. Thus, composites were loaded at a strain rate of 0.2 mm/min (Figure 3).

Tensile tests were performed on the same electronic tensiometer which was used for flexural tests, at a crosshead speed of 0.2 mm/min. An electronic micrometer was utilized to quantify the necessary thickness and width of composite examples. The check length, width and thickness were estimated with a 0.001 mm negligible count mechanized micrometer. This electronic tensiometer was fixed with weight and expansion pointers, which had an insignificant count of 0.01 kg and 0.01 mm, independently. The tensile tests were performed according to ASTM-D3039 testing standards. The specimen dimensions were considered 165 mm × 20 mm × 3 mm (length, width, thickness). In each set of composites, a total of five samples were tested (total 30 measurements).

Impact tests were performed on a falling dart impact testing machine (Impact analyzer M/S International equipment, Mumbai, India). The composite samples were tested as per ASTM D256-97 testing standards, and the specimen dimensions were 63.5 mm long, 12.36 mm wide and 3 mm in thickness. Designed composites were fixed in the vertical position and a pendulum was used to break the composite. The pendulum sled was delivered from the locking position, which is at a state of 1500 mm and hits the sample with a striking rate of 2.46 m/s. The sample was stripped and energy was exhibited in joules by the pointer on the specific scale.

The amount of moisture absorbed by the composite samples when exposed to moisture was analyzed using the moisture analysis test. The tests were performed according to ASTM-D 570 testing standards. The sample specifications were considered as 60 mm long, 20 mm wide and 3 mm in thickness. The samples were placed in a container full of water at room temperature and the amount of moisture gained by the composites was measured for six weeks’ duration (readings taken after every 7 days).

Finally, morphological studies such as surface morphology, porosity content and fracture mechanism were investigated using SEM and the chemical composition of the composite samples was analyzed by using SEM-EDX spectroscopy.

## 3. Results and Discussion

The basalt and E-glass fiber hybrid composites (B, E, B/E, B/E/G5, B/E/G10 and B/E/G15) in the presence of graphite filler (with varying weight fractions 5%, 10% and 15%) were tested to evaluate their mechanical characteristics, wettability and surface morphology.

### 3.1. Wettability Analysis

**Figure 4:**
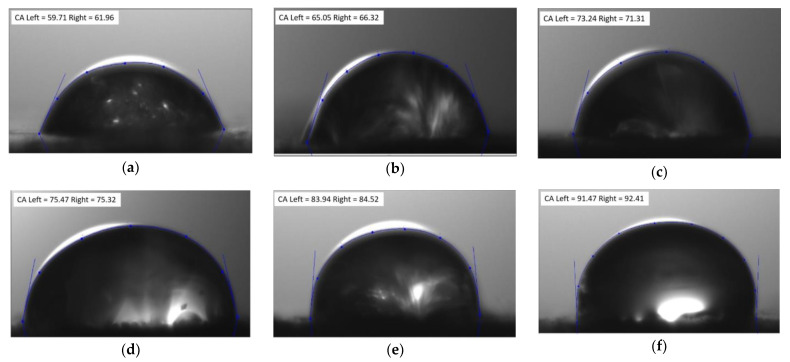
The droplet profile’s polynomial fit was achieved by using knots that go from the left to the right while accounting for the contact angle (**a**) B, (**b**) E, (**c**) B/E, (**d**) B/E/G5, (**e**) B/E/G10 and (**f**) B/E/G15.

The wettability properties of composite materials were investigated using the contact angle measurement technique imaging water droplet images on the composite surface (Figure 4). The volume of the water droplet was kept constant and the values are noted from the left lower endpoint to the right lower endpoint.

**Figure 5:**
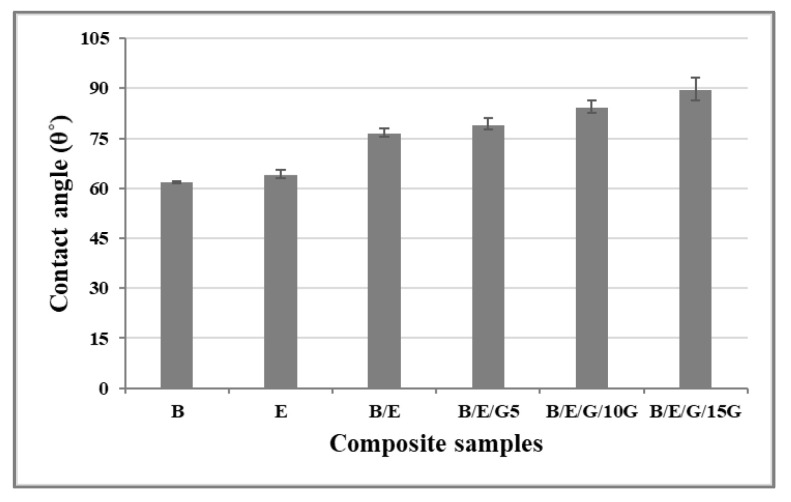
Contact angle measurement for composite samples.

Results (Figure 5) showed the assessment of contact angle measurement of basalt and E-glass woven-fabric hybrid composites and that all sample surfaces exhibited hydrophilic properties. The measured contact angle formed by the water droplet on the B/E/G15 hybrid composite was 86.45° with an error of ±2.36°, being the maximum among all, whereas for individual basalt (B) composites, the least contact angle of 61.76° with an error of ±1.84° was exhibited. Thus, if the contact angle is less than 90°, then it is considered hydrophilic, and if it is more than 90°, then it is hydrophobic [37,38,39]. Moreover, hybrid composites with graphite filler material tended to exhibit hydrophobic surface properties. To extend the analysis, the same tests were conducted varying the measurements in time, and the results showed that the contact angle decreased with an increase in time (at the continuous intervals of time such as 10 s, 20 s, 30 s) in all the cases due to the seeping of a water droplet on the composite surface. The hydrophilic surface properties of the composite material play a prominent role during the formation of micro channels on solid surfaces (composite material).

### 3.2. Flexural Properties

**Figure 6:**
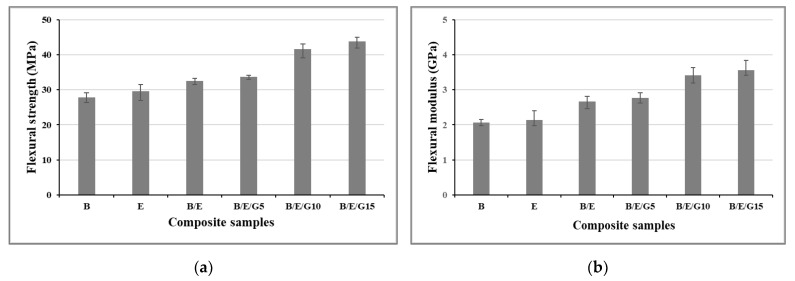
Flexural strength (**a**) and flexural modulus (**b**) of basalt and E-glass composite samples.

**Figure 7:**
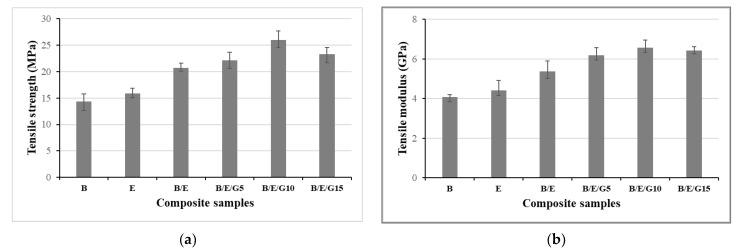
Tensile strength (**a**) and tensile modulus (**b**) of basalt and E-glass composite samples.

The flexural properties, such as the flexural strength and flexural modulus, of basalt and E-glass woven-fabric reinforced with epoxy matrix composites, including filler material (graphite powder particles) were investigated (Figure 6). Results showed that, the strength and modulus were superior in hybrid composites compared to pure composites. The flexural properties increased with filler material and the impact of hybridization was observed, i.e., there was a slight increment in both strength and flexural modulus in basalt and E-glass hybrid composites over pure basalt and pure E-glass woven-fabric composites. The epoxy composites compared to pure basalt (B) composites exhibited the least flexural strength and modulus at 27.82 MPa and 2.06 GPa, respectively. Thus, basalt and E-glass (B/E/G15) hybrid composite with 15% graphite filler material showed the highest values of 43.81 MPa flexural strength and 3.57 GPa flexural modulus (Figure 7). Many researchers found that the incorporation of filler materials into the composite material increased both flexural strength and flexural modulus [40,41]. The reason is attributed to the uniform distribution of filler material and the strong adhesion properties between polymer matrix and filler material. Hence, the composites with graphite filler material exhibited superior flexural strength and modulus properties.

Experimental investigations showed that incorporation of the filler in to different matrices played a crucial role in improving the mechanical performance of the designed novel composite materials.

### 3.3. Tensile Properties

The experiments with basalt and E-glass woven-fabric composites were conducted by performing tensile testing (Figure 7). Thus, the obtained results showed, that the impact of filler material in the hybrid composites was quite noticeable and effective. Unlike the flexural properties, the tensile strength and tensile modulus were increased up to 10% with a graphite filler material. Results for basalt and E-glass (B/E) hybrid composites and basalt and E-glass hybrid composites with 5% graphite particles (B/E/G5) were almost identical. The epoxy-based basalt fiber composite showed the least tensile strength and modulus of 14.68 MPa and 4.07 GPa, respectively (Figure 7a,b). Thus, epoxy-based basalt and E-glass hybrid composites with 10% filler material showed the highest results, i.e., tensile strength of 27.68 MPa (Figure 7a) and tensile modulus of 6.25 GPa (Figure 7b). Hence, the composition in B/E/G10 could act as a very good reinforcing filler in the hybrid composites.

The presence of a filler material in the composites may improve the adhesion properties and also reduce the porosity content. The hybrid composites in the presence of graphite filler material possessed great strength and modulus over the individual fiber composites. Hence, these exceptional properties lead to a potential replacement of other conventional materials used for microfluidics, in the formation of various dimension groves and channels or in absorbent-coated microchannels.

### 3.4. Impact Properties

**Figure 8:**
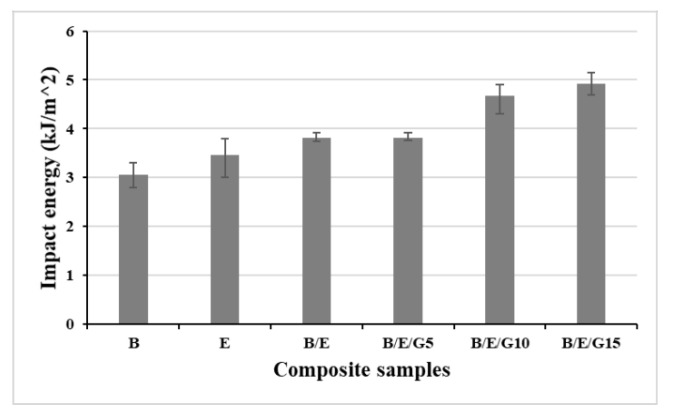
Impact energy of basalt and E-glass composite samples.

**Figure 9:**
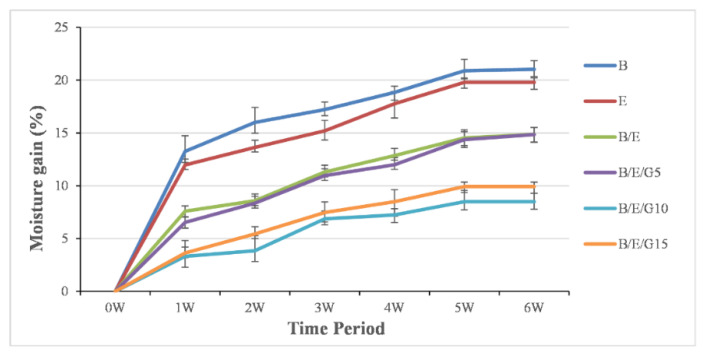
Moisture absorption of basalt and E-glass hybrid composites.

Further, investigations were performed on the impact energy absorbed by the composite samples (Figure 8). It was defined that the amount of energy was absorbed by an object when an external load (impact load or sudden load) was applied to it. Tests were conducted using a Charpy impact tester at room temperature with a relative humidity of 30%. The results followed previously obtained results, i.e., the impact energy increased with an increase in filler material. The pure basalt composites showed the least impact energy at 3.1 kJ/m^2^, and B/E/G15 hybrid composites showed the highest at 4.9 kJ/m^2^. Furthermore, the B/E hybrid composites and B/E/G5 hybrid composites had almost the same impact energy. Composite B/E/G15 exhibited the highest impact energy, and the pure composites showed the lowest impact energy values (Figure 9). This rise in hybrid composites is the result of energy-dissipating fiber-related phenomena such as fiber debonding, pull-out, bridging and fracture, which cause the resin matrix to flex plastically prior to failure. A set of glass fibers with a length greater than the crucial value for effective reinforcement is likely to result in bridging and fiber fractures, while a set of glass fibers with a length less than the critical value is likely to result in debonding and fiber pull-out. Similar to these results, many researchers reported that the hybridization of composites improves the impact strength [42,43]. The same was observed in this research, stating that the hybridization of composites improved the impact strength by two times.

### 3.5. Moisture Analysis

To measure the amount of moisture absorbed by the composites, a moisture analysis test was conducted (Figure 9). After fabrication, the composite samples were placed in an electric oven to eliminate the moisture in them. To analyze the moisture properties, the composite samples were placed in a jar containing distilled water. The readings were noted by differentiating the initial weight and the final weight (after moisture gain) of the composite samples for 6 weeks. After 6 weeks, results showed that the presence of the filler material in a composite sample absorbed less moisture (Figure 9) because the filler material occupied the porosity content in a composite. Thus, pure samples absorbed more moisture than hybrid composites, meaning that the hybrid composites with filler particles have the property of repelling the water when it comes into contact with it. This occurrence was due to unbalanced molecular forces in the water and the solid interface bringing out surface tension. It was observed that the increase in moisture increased over time in all the composites, and after about 5 weeks it reached a steady state.

### 3.6. Morphological Studies

**Figure 10:**
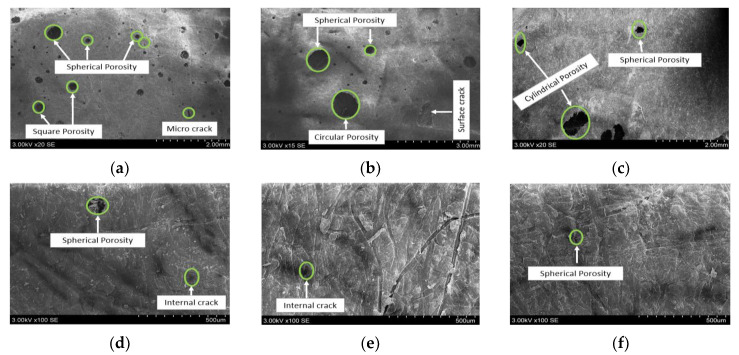
Porosity content in basalt and E-glass woven-fiber hybrid composites (**a**) B, (**b**) E, (**c**) B/E, (**d**) B/E/G5, (**e**) B/E/G10 and (**f**) B/E/G15.

The surface morphology and porosity formation in basalt and E-glass woven-fiber hybrid composites were studied using the SEM. Thus, the presence of pores in a composite may affect the mechanical performance of a composite material. Previous researchers focused on evacuating the air trapped in voids by keeping the volatiles in solution and resin intrusion of the evacuated pore spaces. However, these methods can drive additional problems such as shrinkage and are not suitable for all composites [44,45]. Hence, the graphite powder particles were introduced into the composite material to avoid the microbial effect in it. The SEM results showed that the presence of pores in the form of spherical, cylindrical and helical shapes were observed in the hybrid composites without filler particles (Figure 10a–c). The percentage of porosity in hybrid composites with graphite filler material was smaller when compared to the pure composites (Figure 10d–f). Most of the pores were replaced with nanoparticles in the B/E/G hybrid composites and that can be attributed to improving the mechanical performance and adhesion properties.

**Figure 11:**
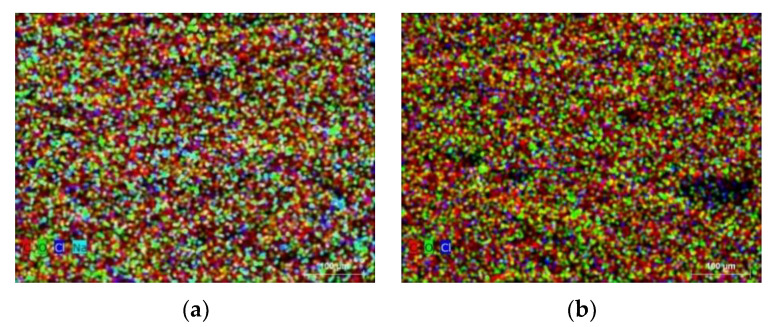

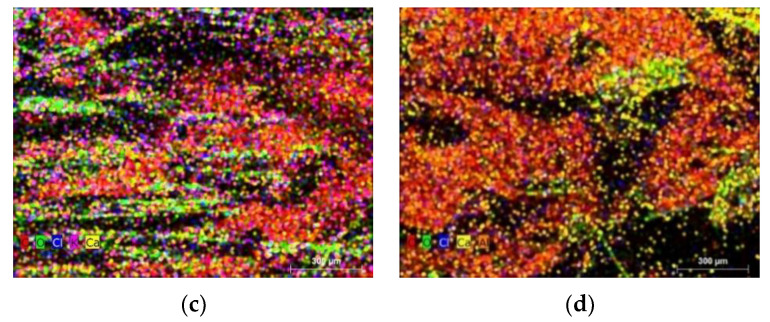
SEM images for map data of hybrid composites (**a**) B/E at surface, (**b**) B/E/G10 at surface, (**c**) B/E at fracture and (**d**) B/E/G10 at fracture.

The morphological studies were conducted on fractured hybrid samples to understand the chemical composition of the composites. It was observed from SEM-EDX results that hybrid composites exhibited good adhesion properties as well as the surface roughness on composite materials (Figure 11). The map data of chemical composition allowed to evaluate the dominant materials in designed composites: the color notations are red for carbon (C), green for oxygen (O), blue for chlorine (Ci), yellow for calcium (Ca), light blue for sodium (Na) and orange for aluminum (Al). The dominance of carbon in the B/E/G10 hybrid composite was observed clearly, i.e., it was more at fracture over the surface position. In the B/E hybrid case, the presence of oxygen on the surface position was observed. Thus, a detailed description of atomic and weight concentrations is given in the Table 3.

**Figure 12:**
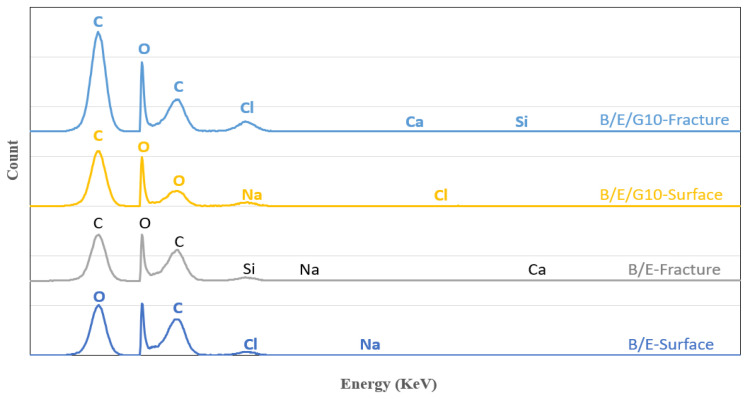
SEM-EDX spectrum analysis on hybrid composites at surface and fracture position.

The chemical composition of basalt and E-glass woven-fabric hybrid composites with and without filler materials was analyzed by using SEM-EDX spectroscopy, too. The chemical composition of a hybrid composite at surface and fracture position was observed for comparison purposes. The highest peak was observed for carbon followed by oxygen. The results stated that the carbon percentage was higher at a fractured place than the surface position (Figure 12).

Due to the presence of graphite particles in B/E/10G, the percentage of carbon reached 78.09% at the fracture position, whereas at the surface position it was 67.72% (Table 3).

Overall, the results proved that the mechanical performance of basalt and E-glass woven-fabric hybrid composites increased with the addition of filler material. The effect of the hybridization concept on woven-fiber laminates was quite impressive. Thus, the designed novel hybrid composites in the presence of filler material can be useful for various engineering applications such as automobiles, construction, biomedical and microfluidics applications. The deeper research into microchannel formation was left for future research.

## 4. Discussion

Novel composite materials with graphite filler were designed for the future design of composite microfluidics chips. Since microfluidics channels are usually made by layering, these channels need to be perfectly aligned on top of each layer. Moreover, the behaviors of fluidic flow mainly dominate because of the interfaces between the fluids and the microchannels’ surface walls, playing critical roles in the regulation of the microflow in the channel. The pressure in microfluidics channel is an important factor, too. Too much pressure in the channel could cause delamination or fracture, depending on the strength of the bonding. Thus, evaluation of surface morphology, wettability and mechanical properties, chemical bonding, etc. are crucial in the design of composites used as the base material for the formation of channels in microfluidics. 

Implementation of novel composite materials opens the opportunity to integrate high-complexity physical and chemical surface modification strategies in the design of microchannels in microfluidics devices. The future work will be focused on the formation of such microchannels using designed composite materials as the basic material, evaluating these materials from the perspective of interfacial interactions between the inner surface and the fluid and determining the behavior of the microflow.

## 5. Conclusions

Basalt and E-glass woven-fiber-based epoxy hybrid composites in the presence of graphite filler material were fabricated using a vacuum-assisted free lamination compression molding fabrication technique.

Novel hybrid composites showed superior mechanical properties over individual basalt and E-glass fiber composites. The addition of filler material to the composite material improved its mechanical performance. It was observed that all the composites exhibited hydrophilic surface properties with a contact angle of less than 90°, and the contact angle increased with an increase in filler material in the composite. From the flexural results, the individual basalt fiber composites exhibited the least flexural strength of 27.82 MPa and flexural modulus of 2.06 GPa. Thus, the B/E/G15 composite showed the highest flexural strength of 43.81 MPa and flexural modulus of 3.57 GPa. From the tensile results, the epoxy-based B/E/10G composites exhibited the highest tensile properties, and epoxy-based basalt composites showed the lower tensile properties. The impact energy values observed in individual basalt and E-glass were 3.1 KJ/m^2^ and 3.5 KJ/m^2^, respectively. The amount of moisture gained by the composites was investigated using moisture analysis. It was observed that all the composites followed the same trend for up to 5 weeks and remained the same even after a 6-week period.

Moreover, the porosity content in hybrid composites with filler material was less than that of composites without filler material. The presence of filler material in hybrid composites showed a sudden peak in carbon weight concentration, proving that incorporation of filler material and the application of the hybridization concept gave superior mechanical performance. Thus, the obtained exceptional composite properties enable future research in the application of microfluidics and microchannels.

## Figures and Tables

**Table 1:** The mechanical properties of materials [33,34,35,36].

Property	Basalt (B)	E-Glass (E)	Epoxy Resin
Density (g/cm^3^)	2.40–2.72	2.48–2.62	1.15–1.18
Diameter (μm)	15	6	-
Tensile Strength (MPa)	4800	3200	72
Young’s Modulus (GPa)	90.0	70.0	2.9
Elongation (%)	3.15	4.00	5.30
Maximum Temp. (°C)	645	450	-
Color	Brown	White	Pale

**Table 2:** Ply sequence and orientation of basalt and E-glass hybrid composites.

Composite	No. of Plys		% Of Graphite Filler Material	Ply Sequence	Orientation (°)
	B	E			
B	6	0	0	B-B-B-B-B-B	0-90-0-90-0-90
E	0	6	0	E-E-E-E-E-E	0-90-0-90-0-90
B/E	3	3	0	B-E-B-E-B-E	0-90-0-90-0-90
B/E/G5	3	3	5	B-E-B-E-B-E	0-90-0-90-0-90
B/E/G10	3	3	10	B-E-B-B-E-E	0-90-0-90-0-90
B/E/G15	3	3	15	B-E-B-E-B-E	0-90-0-90-0-90

**Table 3:** The chemical composition of basalt and E-glass hybrid composites at surface and fracture position.

Element	B/E Surface		B/E Fracture		B/E/G10 Surface		B/E/G10 Fracture	
	Weight (%)	Atom. (%)	Weight (%)	Atom. (%)	Weight (%)	Atom. (%)	Weight (%)	Atom. (%)
Carbon (C)	39.85	48.31	53.56	58.98	67.62	73.24	78.09	81.86
Oxygen (O)	58.77	51.10	45.18	40.57	31.89	26.51	10.85	16.74
Calcium (Ca)	0.00	0.00	0.42	0.14	0.24	0.11	0.08	0.10
Chlorine (Cl)	0.94	0.39	0.00	0.00	0.17	0.08	1.92	1.28
Sodium (Na)	0.44	0.20	0.28	0.13	0.08	0.06	0.00	0.00
Silicon (Si)	0.00	0.00	0.56	0.18	0.00	0.00	0.06	0.02

## Data Availability

Not applicable.
